# Influence of Intrauterine Fluid Detection, Number of Transfers and Age of the Recipient on Pregnancy Rate and Early Embryonic Loss in a Commercial Embryo Transfer Program

**DOI:** 10.3390/ani13111799

**Published:** 2023-05-29

**Authors:** Gian Guido Donato, Denis Necchi, Hilde Vandaele, Micaela Elizabeth Vita, Alessia Bertero, Leila Vincenti, Tiziana Nervo

**Affiliations:** 1 Department of Veterinary Sciences, University of Turin, Largo Paolo Braccini 2, 10095 Grugliasco, Italy; alessia.bertero@unito.it (A.B.); leila.vincenti@unito.it (L.V.); tiziana.nervo@unito.it (T.N.); 2 Keros Embryo Transfer Centre, Westrozebekestraat 23A, 8980 Passendale, Belgium

**Keywords:** mare, subfertility, embryo transfer, early embryonic loss, endometritis, age

## Abstract

**Simple Summary:**

Embryo transfer has become a widely used technology nowadays in the equine breeding industry, and many factors can affect its success. The aim of this study was to determine which recipient factors can affect the pregnancy rate at 14 and 45 days or early embryonic loss. In order to do this, a total of 1222 transfers were analysed. Mares receiving the first embryo of the year had a higher pregnancy rate at 14 and 45 days compared to mares at the third transfer. The detection of intrauterine fluid post ovulation negatively affected the pregnancy rate at 14 days and should therefore be considered an abnormal finding, probably being a sign of uterine inflammation or delayed uterine clearance. Embryo size and grade affected pregnancy rate at 14 and 45 days. On the contrary, the age of the recipient mare and detection of fluid during follicular phase did not affect the pregnancy rate. Only the age of the recipient mare influenced the early embryonic loss, since it was higher in mares aged 10–13 years compared to mares aged 3–5 years. The results of this study can help other embryo transfer facilities in the process of selection of the recipients in order to maximise the efficiency of their embryo transfer program.

**Abstract:**

The selection of the recipient mare is one of the most important factors involved in the success of equine embryo transfer. The aim of this study was to determine whether the age of the recipient, the number of transfers and the detection of intrauterine fluid during the follicular phase or after ovulation can affect pregnancy rate at 14 and 45 days (PR 14 and PR 45) or early embryonic loss (EEL). A total of 1222 ETs were included in the study. Mares receiving the first embryo of the year had a higher PR 14 and 45 days compared to mares at the third transfer (78.8% and 70.1% vs. 65.6% and 54.1%, respectively). The detection of intrauterine fluid post ovulation negatively affected PR 14 (60.5% vs. 77.6%) and should therefore be considered an abnormal finding, probably being a sign of uterine inflammation or delayed uterine clearance. On the contrary, the age of the recipient mare and detection of fluid during follicular phase did not affect PR 14. Only the age of the recipient mare influenced the EEL, since mares aged 10–13 years had a higher EEL compared to mares aged 3–5 years (15.6% vs. 6.4%). Embryo size and grade affected PR 14 and 45.

## 1. Introduction

The first successful attempts of equine embryo transfer (ET) dates back to the mid 1970s [1,2], and currently, ET has become a widely used technology, allowing breeders to produce multiple foals from the same mare in one breeding season, to obtain pregnancies from older mares that had reproductive problems or to obtain foals from mares that are in competition, late-foaling mares, and 2-year-old mares [3,4,5,6]. It has been estimated by the International Embryo Transfer Society that 44,906 embryo flushes and 26,145 transfers of fresh embryos were performed on horses worldwide in 2020 [7]. The pregnancy rate (PR) after transfer can range between 65% and 84%, with an incidence of early embryonic loss (EEL) before 50 days from 10 to 15.5% [8,9,10,11,12,13,14].

The age is an important factor related to fertility in the mare. In older mares, PR tends to decrease, and EEL tends to increase [15,16,17,18,19]. Therefore, it is usually suggested to consider only mares that are 3 to 10–12 years old to be admitted in the recipient herd [6,20,21], even if older recipients have also been used in previous studies [4,9].

Regarding the number of transfers performed on a single recipient during the same breeding season, it is generally considered that a healthy recipient can receive two or three embryos in a year [6]. Within three transfers, this parameter does not seem to affect PR or EEL according to previous studies, since non-surgical transfer technique is not a harmful procedure and does not affect the fertility of the recipient if she does not become pregnant [9,22,23].

Furthermore, since the advent of ultrasound, the detection of intrauterine fluid (IUF) has been reported both in oestrus and dioestrus, and the awareness of the frequency of this abnormality has increased [24,25]. A small quantity of anechoic IUF is a common finding within the uterine lumen of healthy mares during oestrus [26] and generally is considered not to affect pregnancy rates, while a fluid volume exceeding 2 cm has been associated with decreased pregnancy rates [27,28,29]. Even small accumulations of anechoic IUF during the immediate post ovulation period could possibly adversely affect fertility [26], and it has been shown that free fluid within the uterine lumen during dioestrus should be considered abnormal because it indicates an inflammatory process and/or impaired uterine clearance [30]. In fact, inadequate uterine drainage is a cause of subfertility in old pluriparous mares and in some maiden mares that do not completely dilate their cervix during oestrus [27]. However, to the best of the authors’ knowledge, the effect of the detection of IUF in recipient mares before and after ovulation on PR and EEL in an ET program has never been described.

Although selection and management of the recipients are essential for the success of any ET program, other factors related to the embryo and to the donor mare can also affect the results. Among these, the most important are the size, age and grade of the embryo as well as the age of the donor mare [6,8,9].

The aim of this study was to determine if different recipient factors, such as age, number of transfers and detection of intrauterine fluid (IUF) during the follicular phase and after ovulation, affected the pregnancy rate and/or early embryonic loss in a commercial embryo transfer program. The following were hypothesized: (1) Older recipients have a reduced PR and/or increased EEL. (2) Mares at the third transfer of the season have a reduced PR. (3) The presence of IUF before ovulation is not pathological and does not lead to reduced PR or EEL. (4) Detection of IUF post ovulation, being a sign of impaired uterine clearance and/or endometrial inflammation, is associated with reduced fertility.

## 2. Materials and Methods

This study was performed retrospectively based on data of 1222 transfer performed at Keros Embryo Transfer Centre, Passendale (Belgium) during the 2021 and 2022 breeding seasons.

### 2.1. Donor Mares and Embryo Flush

Donor mares’ insemination and flushing for embryo recovery were performed at the centre or at distant locations. In this case, embryos were shipped to the centre in different holding media and cooling devices, according to the practitioners’ preferences.

For the mares inseminated at the centre, after the detection of a follicle larger than 35 mm and the presence of endometrial oedema, 1500 IU of Human Chorionic Gonadotropin (Chorulon^®^, MSD Animal Health SRL, Boxmeer, The Netherlands) were administered intravenously to induce ovulation. Artificial insemination with fresh and chilled semen was performed 24 h after ovulation induction. If ovulation did not occur, mares were inseminated again 48 h after the first AI. When frozen semen was used, insemination was performed within 6 h after the ovulation. The stallions used for insemination were recorded. Donor mares showing fertility problems were treated accordingly.

Uterine flushes for embryo recovery were performed between days 8 and 9 post ovulation (after the first ovulation for mares with multiple ovulations). For flushes performed at the centre, the procedure is briefly described. The rectum was gently emptied, the tail was wrapped and the perineum was washed three times with water and povidone iodine, then dried with paper towel. The uterus was flushed using a three-way silicon tubing system connected to a silicon cuffed 36 CH Bivona catheter (IMV Technologies, L’Aigle, France) and an EZWay Filter (EZ-Way, A&E Int’l, Alta Vista, KS, USA). The catheter was inserted transcervically into the uterus. Once into the uterine body, a cuff on the end of the catheter was inflated with 40–60 mL of air and then pulled caudally to ensure a tight seal against the internal os of the cervix. Donors were flushed three times with 1 to 3 L of a commercial Lactated Ringer’s solution (Baxter SpA, Rome, Italy) depending on the uterus size. After the second and third flush, in order to maximise the recovery of the fluid, the uterus was massaged transrectally. Finally, the air cuff was deflated, and the catheter was removed from the mare. Then, 2 mg of Alfaprostol (Gabbrostim, CEVA, Libourne, France) or 250 mcg of Cloprestenol (Estrumate, MSD-Animal Health SRL, Rahway, NJ, USA) were injected IM to the donor mares in order to induce luteolysis, prevent unwanted pregnancies and accelerate the return in oestrus.

Embryos were searched, graded [6] and measured using a stereomicroscope (Olympus SZX10, Olympus Corporation, Tokyo, Japan) equipped with an eyepiece micrometre, then washed three times in Embryo Holding Medium (IMV Technologies, L’Aigle, France).

### 2.2. Recipient Mares and ET

Recipient mares, aged 3 to 17 years (average: 9.02 ± 3.30; median: 9 years), were housed in single or shared stalls and fed with hay and corn silage; water was supplied ad libitum. In 157 cycles, the recipient was maiden, while in 1013 cycles, the recipient was pluriparous. Only on a few occasions (*n* = 15) was the recipient a lactating mare with foal on foot.

During the follicular phase, the recipients were submitted every two days to transrectal palpation and ultrasound examination in order to detect the ovulation. Then, 4 to 5 days after the detection of ovulation, transrectal palpations and ultrasound examinations were performed, and only mares with a good uterine and cervical tone, absence of uterine oedema, absence of IUF accumulation and presence of a morphologically normal CL were selected to receive an embryo. If the mare was not used as a recipient, 6 to 8 days after the detection of ovulation, 250 mcg of Cloprostenol (Estrumate, MSD Animal Health SRL, Boxmeer, The Netherlands) were administered IM to induce luteolysis, and the mare was scanned 3 to 4 days later.

If intrauterine fluid accumulation was observed during the follicular phase or the day of detection of ovulation (therefore indicating the presence of intrauterine fluid within 0 and 48 h after ovulation), the data were recorded. However, a given mare was used as a recipient in that cycle only if IUF was not present anymore the day of the transfer or the day before. If the fluid detected in any moment of the cycle was more than grade I (anechoic) [24] and >2 cm, the recipient mare was removed from use.

Mares that did not get pregnant after the first transfer received a second embryo or a third embryo during the breeding season, and the data were recorded.

The recipient mares, synchronised alongside the donors, received an embryo between 4 and 7 days post ovulation. The mares were held in a palpation stock and sedated with 0.01–0.02 mg/kg of detomidine hydrochloride IV (Medesedan, Virbac Belgium SA, Leuven, Belgium) alone or in association with butorphanol (Dolorex, MSD Animal Health SRL, Boxmeer, The Netherlands) (0.01–0.03 mg/kg IV) if needed. The embryo was loaded into a 0.25 mL or 0.5 mL straw (depending on the size of the embryo) and the straw loaded into a stainless steel Cassou gun (IMV Technologies, L’Aigle, France), covered by a disposable sterile sheath and an outer sterile chemise (IMV Technologies, L’Aigle, France). All the transfers were performed by the same veterinarian with the “double glove” manual technique. Briefly, the tip of the instrument was held by a hand wearing a sterile sleeve and covered by a second sterile obstetrical sleeve and introduced in the cervix. Once the tip was inserted in the cervical os, the chemise was broken and pulled caudally. Subsequently, the operator used transrectal manipulation to ensure that the pipette entered the uterus, minimising manipulation of the cervix, and finally the embryo was deposited into the uterus, as deep as possible into one uterine horn. Before or after the transfer the recipients did not receive any treatment.

Transrectal ultrasound examination (MyLabOne, Esaote, Italy) was performed 4 to 6 days after ET in order to detect the pregnancy (these data indicate PR at 14 days). Subsequently, pregnancy examinations were performed every one to two weeks, and a final one was performed between 45 and 50 days after ET (PR at 45 days). Early embryonic loss (EEL) was calculated as the number of embryo losses between the first and last pregnancy check, divided by the number of mares pregnant at the first pregnancy check.

### 2.3. Statistical Analysis

Data were analysed using the software R 4.2.2. Three generalised linear models were created to observe the effect of different factors on PR at 14 and 45 days and EEL (dependent variables). For all the models, the factors investigated were as follows: (1) age of the recipient mare (4 groups: 3–5 years, 6–9 years, 10–13 years, 14–17 years), (2) number of transfers during the season on the same recipient, (3) detection of fluid during the follicular phase, (4) detection of fluid after ovulation, (5) embryo size (4 groups: <300 µm, 300–600 µm, 601–1500 µm, >1500 µm) and (6) embryo quality grade (3 groups: grade I, grade II, grade III). For data showing differences < 0.05, Pearson’s Chi-squared test, applying Bonferroni’s correction in the case of multiple comparisons, was used to compare differences between levels. Pearson’s Chi-squared test was used to observe the relation between detection of IUF before and after ovulation and age. Furthermore, Pearson’s Chi-squared test was used to observe the influence of the stallion on PR14, PR45 and EEL; in order to do this, only stallions with more than 30 embryos each were selected.

## 3. Results

An overall PR at 14 days of 77.1% was achieved and EEL had an incidence of 11.7%. PR at 45 days was 68.1%. The factors that had a statistically significant effect on PR 14 were number of transfers (*p* = 0.045), detection of fluid post ovulation (*p* = 0.02), embryo size (*p* < 0.01) and grade (*p* < 0.001). Age of the recipient mare and detection of fluid before ovulation were not significant. Only the age of the recipient mare influenced the EEL (*p* = 0.032), while number of transfers (*p* = 0.03), embryo size (*p* < 0.001) and grade (*p* < 0.001) influenced PR 45 (Table 1).

The age of the recipient mare was recorded for 1206 transfers. The mares were divided into four age groups: 3–5 years, 6–9 years, 10–13 years and 14–17 years. This factor did not influence the PR at 14 or 45 days but affected the EEL, since mares in the 3–5 years old group had a lower EEL than mares between 10 and 13 years (6.4% and 15.6%, respectively, *p* = 0.03).

Regarding the number of transfers, 893 transfer were performed on recipient mares at the first transfer of the season, 252 on mares at the second transfer and 61 on mares at the third transfer. There are no differences between mares receiving one or two embryos in one year (PR 14 = 78.8% and 75.4%; *p* > 0.1; PR 45 = 70.1% and 66.7%; *p* > 0.1, respectively), but the PR 14 and 45 of the third transfer of the season is significantly lower compared to the first one (PR 14 = 65.6%, *p* = 0.04; PR 45 = 54.1%, *p* = 0.026).

Grade I (anechoic) [24] and <2 cm fluid during the follicular phase was detected in 155 cycles (12.7%). The pregnancy rate at 14 or 45 days was not statistically different between recipient mares with IUF and mares without (respectively PR 14 = 74.8% and 77.4%, *p* > 0.1; PR 45 = 63.2% and 69%, *p* > 0.1) and EEL was also not affected (*p* > 0.1). Detection of fluid during the follicular phase was not related to the age of the recipient (*p* > 0.1).

Grade I (anechoic) [24] and <2 cm fluid after ovulation was observed in 38 cycles (3.1%) and negatively affected the PR 14 (60.5% in mares with fluid vs. 77.6% in mares without fluid, *p* = 0.013), but not the EEL or PR 45 (*p* > 0.1). The fluid was detected mostly in young (3–5 years, 6.5%) and old mares (14–17 years, 7.3%), compared to the other two groups (6–9 and 10–14 years, respectively, 2.6% and 1.2%; *p* < 0.001).

A given mare was not used as embryo recipient in that cycle because intrauterine fluid was detected the day of transfer or the day before on 53 occasions.

The size of the embryo affected PR 14 and PR 45. Embryos <300 µm had a lower PR 14 than embryos between 300 and 600 µm (67.4% and 78.2%, respectively, *p* = 0.047) and between 601 and 1500 µm (79.6%, *p* = 0.026). Regarding PR at 45 days, it was significantly lower in embryos <300 µm than in embryos between 300 and 600 µm (55.6% and 69.5%, respectively, *p* = 0.01) and between 601 and 1500 µm (72.1%, *p* < 0.01). PR 45, moreover, was lower in embryos >1500 µm than in embryos between 601 and 1500 µm (51.3%, *p* = 0.04).

The grade of the embryo influenced both PR 14 and PR 45, but not EEL. Grade I embryos had a statistically significant higher PR 14 than grade II (78.8% vs. 63.7%, respectively, *p* < 0.001) and grade III (38.9%, *p* < 0.0001). PR 45 was higher in grade I embryos than grade II or III (70.1%, 55% and 38.9%, respectively, *p* = 0.015 and 0.01).

Finally, considering the stallion factor, a total of 287 different stallions were used in this study. Only stallions that produced at least 30 embryos were included in this analysis, therefore 5 stallion were included. However, PR 14, PR 45 and EEL were not statistically different between the 5 stallions (*p* > 0.1) (range PR 14: 72–85.7%; range PR 45: 62–76.9%; range EEL: 4.3–13.9%).

## 4. Discussion

Hypothesis 1 that PR and EEL are influenced by the age of the recipient was partially supported by this study. The PR at 14 and 45 days was not different between mares belonging to different age groups, but EEL was increased in mares between 10 and 13 years compared to very young mares aged between 3 and 5 years. This result is in accordance with Carnevale et al. [9], who observed no differences in PR between younger (2–9 years) and older recipients (10–18 years) but noticed a tendency to an increase in EEL in the group of older mares (13.3% vs. 20.5%). Cuervo-Arango et al. and Claes et al. [11,31], on the other hand, found out that age of the recipient mare did not affect the likelihood of pregnancy and embryonic loss after transfer of fresh and cryopreserved in vitro produced equine embryos. If we look at studies regarding mares that are not subjected to ET but are inseminated and carry their own pregnancy, the increase of age is associated with a decrease of PR and an increase of EEL [15,16,17,18,32,33,34]. However, in our study we did not observe differences in PR at 14 days in older recipients compared to the younger ones. Probably, part of the explanation for this result is that many factors related to age differently affect PR at 14 days in recipient mares compared to mares that are inseminated. In fact, older mares that are inseminated usually experience poor quality and reduced viability of oocytes and embryos, poor oviductal and uterine environment and inflammation as a response to insemination [34,35,36,37,38,39,40,41]. These age-related factors play an important role in the process of fertilisation and early embryo developing and significantly affect PR 14 in these mares. On the contrary, since fertilisation and the first phases of the embryo developing do not occur in recipient mares, the same age-related factors are not involved in determining pregnancy rate, and this could explain why this outcome was not statistically different in younger and older recipients. Furthermore, it is important to highlight that the recipients in this ET program are not randomly selected and representative of the population of older mares but are subjected to a selection based on fertility, and only recipients that successfully got pregnant after a maximum of three transfers are maintained in the recipient herd. Therefore, mares in the older group are recipients that did not show any fertility problem throughout their reproductive career, while subfertile mares were eliminated.

On the other hand, endometrial degenerative changes, which are related to age and parity, have an important influence on recipients’ EEL and are probably the cause of its increase in one group [42,43,44]. It is interesting to notice that the higher incidence of EEL was observed in mares between 10 and 13 years old and not in the oldest group (>14 years). The possible explanation is that the increase of EEL between 10 and 13 years witnesses the moment when fertility starts to decline [15,16,17], but the strict selection on recipients eliminates the effect of age in the older group, since only the most fertile mares are kept in the herd.

Hypothesis 2, stating that mares at the third transfer of the season have a lower PR, was substantiated by the results of this study. Previously, different authors concluded that there are no differences in PR between mares that received one, two or three embryos during the same season [9,45]. However, in our study, we observed a decreased PR at 14 and 45 days in mares at the third transfer compared to mares at the first one. No differences were found between mares at the first and second transfer, probably because in most cases, the first failure was due to the poor quality of embryos or other extrinsic factors not related to a reduced fertility of the mare, and, therefore, mares after one failure can be safely used as embryo recipients for a second time. Most of the mares that already have two previous failures and received a third transfer, on the contrary, probably had a non-optimal uterine environment or other reproductive abnormalities that led to a reduced PR. Therefore, mares after two failures should be used for a third transfer only if strictly necessary.

Hypotheses 3 and 4 are based on whether the detection of IUF in the recipient mares in different moment of the oestrus cycle has a clinical significance, affecting PR or EEL, and should therefore be considered a parameter to disqualify a mare from receiving an embryo in that cycle. IUF is one of the symptoms associated with delayed uterine clearance and endometritis and is a useful tool in the diagnosis of this pathology [46]. However, the relationship between the presence of fluid, the moment of the cycle when fluid is detected and the success of transfer has never been studied in the recipient mare.

Hypothesis 3, stating that the presence of a IUF accumulation <2 cm during the follicular phase is not pathological and does not lead to a reduced PR and EEL, was supported by the results of this study. Intrauterine fluid originates from secretions of endometrial glands, from transudation and from failure of the mechanical uterine clearance [47]. Detection of small quantities of IUF during the follicular phase does not necessarily indicate inflammation, but it can also be transudate from the vessels, associated with the same mechanism that causes normal oestrous oedema; in fact, oestrogens increase the permeability of the endothelium of uterine vessels, endometrial secretion and oedema during oestrus [47,48,49,50]. Furthermore, in many cases, the IUF detected during oestrus is sterile and does not contain inflammatory cells [48,50]. However, the amount of fluid that should be considered significant is not clear, but small quantities during oestrus in mares that are inseminated usually do not affect PR [46,50,51], in contrast to larger (>2 cm) collections of fluid [24,27,28,48]. Regarding recipient mares, even if the presence of IUF during oestrus is generally considered an important factor in the selection [6], our study confirmed the hypothesis that the presence of an anechoic IUF accumulation <2 cm during oestrus is not a pathological finding and does not negatively affect PR and EEL.

The results of this study also partially supported hypothesis 4, since the detection of IUF accumulation in the recipient mare after ovulation was related to a decreased PR at 14 days. It has to be considered, however, that the depth of IUF accumulation (except for being <2 cm) was not evaluated, and this is a limitation of the present study. This hypothesis relies on the assumption that even a small quantity of intraluminal fluid during the immediate post ovulation period and during dioestrus is reported to be a clinical sign of inflammation, which identifies mares that are inefficient at mechanically evacuating the uterus and is related to subfertility [26,48,49]. The detection of IUF after ovulation (between 0 and 2 days) in our study indicated mares that failed to clear the uterus or accumulated fluid after the ovulation. Inadequate uterine clearance is described as a cause of subfertility in aged pluriparous mares and in some old and young maiden mares that do not completely relax their cervix during oestrus [27], and this result was confirmed by our study, since fluid was observed mainly in the group of very young (3–6 years) and old recipients (14–17 years). Furthermore, in a previous study, it was observed that the incidence of intrauterine fluid during dioestrus was associated with the presence of an inflammatory process, as indicated by a high biopsy score, reduced progesterone concentrations and a shorter inter-ovulatory interval, resulting in a lower PR and higher EEL [30,46]. Cytological, bacteriological or histological examination to diagnose uterine inflammation and therefore endometritis have not been performed due to the retrospective nature of this study, but the presence of IUF in the early luteal phase could possibly represent a sign of endometritis, which could explain the reduction of PR. Endometritis represents an important factor to be considered in the management of recipient mares that can significantly affect the success of ET [20]. It is a multifactorial disease, and physical clearance through the cervix plays an important role in the resistance of mares to uterine infection, since it has been demonstrated that mares susceptible to endometritis have a delayed uterine clearance [47,52]. Furthermore, IUF accumulations provide an appropriate environment for the growth of bacteria [50].

In ET programs, recipients are generally scanned on the day of the transfer to assess, among other parameters, the absence of IUF [6]; in fact, the presence of small amounts of fluid at this time decreases the success of embryo transfer [53]. On the other hand, the correlation between the presence of fluid in the first two days after ovulation and the success of ET has never been investigated. It is recommended by some practitioners to scan recipients for at least one day post ovulation to assess that there is no fluid in the uterus as they enter dioestrus [20], due to the possible detrimental effect on fertility of this finding. However, this is the first study that confirms this hypothesis and describes a negative correlation between the detection of IUF after ovulation and PR in recipient mares in a commercial ET program.

Finally, although the main aim of the study was to observe which recipient factors can influence the success of ET (hypothesis 1–4), we had to include and consider other factors that may have had a significant impact on the results. These factors were related to the embryo (size and grade) and to the stallion. Regarding the embryo, we observed that small embryos had a lower PR 14 and 45. Similar results have been already described by different authors; probably, the reason underlying the decreased success is not the small size per se, but because they are “small for age”, showing a delay in the development [6,54]. Furthermore, we noticed that also larger embryos have a lower PR at 45 days, and this could probably be explained because they are more fragile and could be more easily damaged during handling or transfer [6,55]. The embryo grade also influenced the success of ET in our study, since grade II and III embryos had significantly lower PR 14 and 45 compared to grade I embryos, as previously reported by other papers [8,9,54]. Regarding the stallions, this parameter did not affect the outcome of ET, since PR and EEL were not statistically different for embryos produced from five different stallions. On the other hand, it has been shown by several studies that embryos from old and subfertile donor mares have lower PR and/or higher EEL than those from young and healthy mares, probably due to lower quality oocytes and/or to an impaired uterine or oviductal environment [37,40,56,57]. However, a limitation of the present study is that age and fertility of the donor mare were not evaluated. Furthermore, another limitation of the present study was that the influence of shipment and factors associated to shipment (temperature, distance, medium, cooling device) were not evaluated, even if, according to different authors, it seems that there is no difference in the outcome of the transfer of transported embryos compared to the fresh ones [5,6,9,58]

## 5. Conclusions

In conclusion, the detection of IUF after ovulation negatively affected PR at 14 days, while the number of transfers, embryo size and grade affected PR at both 14 and 45 days. Age of the recipient and detection of IUF during the follicular phase did not influence PR. Only the age of the recipient was able to affect EEL, since older mares (10–13 years) showed a higher EEL than younger ones (3–5 years). The results of this study can help other ET facilities in the process of selection of the recipients, in order to maximise the efficiency of the ET program.

## Figures and Tables

**Table 1:** Different factors affecting pregnancy rate and early embryonic loss. Within a column, different letters (a,b,c) indicate a significant difference (*p* < 0.05).

	PR 14	EEL	PR 45
Age			
3–5 yo (*n* = 184)	76.6% a	6.4% a	71.7% a
6–9 yo (*n* = 499)	77.2% a	11.2% a,b	68.5% a
10–13 yo (*n* = 414)	79% a	15.6% b	66.7% a
14–17 yo (*n* = 109)	71.6% a	9% a,b	65.1% a
Number of transfers			
1 (*n* = 893)	78.8% a	11.1% a	70.1% a
2 (*n* = 252)	75.4% a,b	11.6% a	66.7% a,b
3 (*n* = 61)	65.6% b	17.5% a	54.1% b
Iuf during oestrus			
Yes (*n* = 155)	74.8% a	15.5% a	63.2% a
No (*n* = 1067)	77.4% a	10.9% a	69% a
Iuf after ovulation			
Yes (*n* = 38)	60.5% a	8.7% a	55.3% a
No (*n* = 1184)	77.6% b	11.9% a	68.4% a
Embryo size			
<300 µm (*n* = 135)	67.4% a	17.6% a	55.6% a
300–600 µm (*n* = 609)	78.2% b	11.1% a	69.5% b,c
601–1500 µm (*n* = 377)	79.6% b	9.3% a	72.1% b
>1500 µm (*n* = 39)	64.1% a,b	20% a	51.3% a,c
Embryo grade			
I (*n* = 1092)	78.8% a	11% a	70.1% a
II (*n* = 80)	63.7% b	13.7% a	55% b
III (*n* = 18)	38.9% b	0% a	38.9% b

## Data Availability

Data sharing is not applicable.

## References

[1] Allen W., Rowson L. (1975). Surgical and Non-Surgical Egg Transfer in Horses. J. Reprod. Fertil. Suppl..

[2] Oguri N., Tsutsumi Y. (1974). Non-Surgical Egg Transfer in Mares. J. Reprod. Fertil..

[3] Squires E.L. (2019). Perspectives on the Development and Incorporation of Assisted Reproduction in the Equine Industry. Reprod. Fertil. Dev..

[4] Iuliano M., Squires E. (1985). Embryo Transfer in Two-Year-Old Donor Mares. Theriogenology.

[5] Squires E. (2020). Current Reproductive Technologies Impacting Equine Embryo Production. J. Equine Vet. Sci..

[6] McCue P., Squires E. (2015). Equine Embryo Transfer.

[7] Viana J.H.M. 2020 Statistics of Embryo Production and Transfer in Domestic Farm Animals. https://www.iets.org/Portals/0/Documents/Public/Committees/DRC/IETS_Data_Retrieval_Report_2020.pdf.

[8] Panzani D., Vannozzi I., Marmorini P., Rota A., Camillo F. (2016). Factors Affecting Recipients’ Pregnancy, Pregnancy Loss, and Foaling Rates in a Commercial Equine Embryo Transfer Program. J. Equine Vet. Sci..

[9] Carnevale E.M., Ramirez R.J., Squires E.L., Alvarenga M.A., Vanderwall D.K., Mccue P.M. (2000). Factors Affecting Pregnancy Rate and Early Embryonic Death after Equine Embryo Transfer. Theriogenology.

[10] Cuervo-Arango J., Claes A.N., Stout T.A.E. (2019). Small Day 8 Equine Embryos Cannot Be Rescued by a Less Advanced Recipient Mare Uterus. Theriogenology.

[11] Cuervo-Arango J., Claes A.N., Ruijter-Villani M., Stout T.A. (2018). Likelihood of Pregnancy after Embryo Transfer Is Reduced in Recipient Mares with a Short Preceding Oestrus. Equine Vet. J..

[12] Fanelli D., Tesi M., Ingallinesi M., Camillo F., Panzani D. (2022). Recipients’ Pregnancy Rate Was Affected by Season but Not by the Temperature-Humidity Index (THI) in an Equine Commercial ET Programme in Southern Europe. Reprod. Domest. Anim..

[13] Riera F. (2011). General Techniques and Organization of Large Commercial Embryo Transfer Programs. Clin. Theriogenology.

[14] Okada C.T.C., Segabinazzi L.G., Crespilho A.M., Dell’Aqua J.A., Alvarenga M.A. (2018). Effect of the Flunixin Meglumine on Pregnancy Rates in an Equine Embryo Transfer Program. J. Equine Vet. Sci..

[15] Allen W.R., Brown L., Wright M., Wilsher S. (2007). Reproductive Efficiency of Flatrace and National Hunt Thoroughbred Mares and Stallions in England. Equine Vet. J..

[16] Nath L.C., Anderson G.A., McKinnon A.O. (2010). Reproductive Efficiency of Thoroughbred and Standardbred Horses in North-East Victoria. Aust. Vet. J..

[17] Scoggin C.F. (2015). Not Just a Number: Effect of Age on Fertility, Pregnancy and Offspring Vigour in Thoroughbred Brood-Mares. Reprod. Fertil. Dev..

[18] Bosh K.A., Powell D., Shelton B., Zent W. (2009). Reproductive Performance Measures among Thoroughbred Mares in Central Kentucky, during the 2004 Mating Season. Equine Vet. J..

[19] Marinone A.I., Losinno L., Fumuso E., Rodríguez E.M., Redolatti C., Cantatore S., Cuervo-Arango J. (2015). The Effect of Mare’s Age on Multiple Ovulation Rate, Embryo Recovery, Post-Transfer Pregnancy Rate, and Interovulatory Interval in a Commercial Embryo Transfer Program in Argentina. Anim. Reprod. Sci..

[20] Hartman D.L., McKinnon A.O., Squires E.L., Vaala W.E., Verner D.D. (2011). Embryo Transfer. Equine Reproduction.

[21] Squires E.L., Mccue P.M., Vanderwall D. (1999). The current status of equine embryo transfer. Theriogenology.

[22] Riera F. Systematic Approach to Efficiency Problems in an Embryo Transfer Program. Proceedings of the SFT—Theriogenology Annual Conference.

[23] Riera F.L., McDonough J. (1993). Commercial Embryo Transfer in Polo Ponies in Argentina. Equine Vet. J..

[24] Pycock J.F., Mc Kinnon A.O., Squires E., Vaala W., Varner D. (2011). Treatment of Fluid Accumulation. Equine Reproduction.

[25] Allen W., Pycock J. (1988). Cyclical Accumulation of Uterine Fluid in Mares with Lowered Resistance to Endometritis. Vet. Rec..

[26] Brinsko S., Blanchard T.L., Varner D., Schumacher J., Love C.C. (2011). Manual of Equine Reproduction.

[27] LeBlanc M., Mc Kinnon A., Mc Kinnon A., Squires E., Vaala W., Varner D. (2011). Breeding the Problem Mare. Equine Reproduction.

[28] Brinsko S.P., Rigby S.L., Varner D.D., Blanchard T.L. (2003). A Practical Method for Recognizing Mares Susceptible to Post-Breeding Endometritis. Proc. Am. Assoc. Equine Pract..

[29] Freccero F., Mislei B., Bucci D., Dondi F., Mari G. (2022). Effects of Intra-Uterine Fluid Accumulation after Artificial Insemination on Luteal Function in Mares. Animals.

[30] Adams G.P., Kastelic J.P., Bergfelt D.R., Ginther O.J. (1987). Effect of Uterine Inflammation and Ultrasonically-Detected Uterine Pathology on Fertility in the Mare. J. Reprod. Fertil. Suppl..

[31] Claes A., Cuervo-Arango J., van den Broek J., Galli C., Colleoni S., Lazzari G., Deelen C., Beitsma M., Stout T.A. (2019). Factors Affecting the Likelihood of Pregnancy and Embryonic Loss after Transfer of Cryopreserved in Vitro Produced Equine Embryos. Equine Vet. J..

[32] Baker C.B., Little T.V., McDowell K.J. (2010). The Live Foaling Rate per Cycle in Mares. Equine Vet. J..

[33] Morris L.H.A., Allen W.R. (2002). Reproductive Efficiency of Intensively Managed Thoroughbred Mares in Newmarket. Equine Vet. J..

[34] Catandi G.D., Obeidat Y.M., Broeckling C.D., Chen T.W., Chicco A.J., Carnevale E.M. (2021). Equine Maternal Aging Affects Oocyte Lipid Content, Metabolic Function and Developmental Potential. Reproduction.

[35] Barbacini S., Necchi D., Zavaglia G., Squires E.L. (2003). Retrospective Study on the Incidence of Postinsemination Uterine Fluid in Mares Inseminated With Frozen/Thawed Semen. J. Equine Vet. Sci..

[36] Woodward E.M., Christoffersen M., Campos J., Squires E.L., Troedsson M.H.T. (2012). Susceptibility to Persistent Breeding-Induced Endometritis in the Mare: Relationship to Endometrial Biopsy Score and Age, and Variations between Seasons. Theriogenology.

[37] Vogelsang S.G., Vogelsang M.M. (2010). Influence of Donor Parity and Age on the Success of Commercial Equine Embryo Transfer. Equine Vet. J..

[38] Carnevale E.M., Griffin P.G., Ginther O.J. (1993). Age-Associated Subfertility before Entry of Embryos into the Uterus in Mares. Equine Vet. J..

[39] Rambags B.P.B., van Boxtel D.C.J., Tharasanit T., Lenstra J.A., Colenbrander B., Stout T.A.E. (2014). Advancing Maternal Age Predisposes to Mitochondrial Damage and Loss during Maturation of Equine Oocytes Invitro. Theriogenology.

[40] Ball B.A., Little T.V., Weber J.A., Woods G.L. (1989). Survival of Day-4 Embryos from Young, Normal Mares and Aged, Subfertile Mares after Transfer to Normal Recipient Mares. J. Reprod. Fertil..

[41] Pinto-Bravo P., Reborddo M.R., Amaral A., Fernandes C., Cuello C., Parrilla I., Martínez E., da Costa R.P.R., Skarzynski D.J., Ferreira-Dias G. (2018). Is Mare Endometrosis Linked to Oviduct Fibrosis?. Pferdeheilkunde.

[42] Carnevale E.M., Ginther O.J. (1992). Relationship of Age to Uterine Function and Reproductive Efficinecy in Mares. Theriogenology.

[43] Esteller-Vico A., Liu I.K., Couto S. (2012). Uterine Vascular Degeneration Is Present throughout the Uterine Wall of Multiparous Mares. Colinearity between Elastosis, Endometrial Grade, Age and Parity. Theriogenology.

[44] Dubrovskaya A.B., Lebedeva L.F., Schukis K.A. (2019). Comparative Histomorphological Characteristics of the Endometrium of Young and Aged Mares in Estrus and Diestrus. IOP Conf. Ser. Earth Environ. Sci..

[45] Riera F.L., Samper J.C. (2009). Equine Embryo Tranfer. Equine Breeding Management and Artificial Insemination.

[46] McKinnon A.O., McCue P.M., McKinnon A.O., Squires E.L., Vaala W.E., Varner D.D. (2011). Uterine Abnormalities. Equine Reproduction.

[47] Katila T., Ferreira-Dias G. (2022). Evolution of the Concepts of Endometrosis, Post Breeding Endometritis, and Susceptibility of Mares. Animals.

[48] Pycock J.F., Newcombe J.R. (1996). The Relationship between Intraluminal Uterine Fluid, Endometritis and Pregnancy Rate in the Mare. Equine Pract..

[49] Pycock J.F., Samper J.C. (2009). Breeding Management of the Problem Mare. Equine Breeding Management and Artificial Insemination.

[50] Reilas T., Katila T., Makela O., Huhtinen M., Koskinen E. (1997). Intrauterine Fluid Accumulation in Oestrous Mares. Acta Vet. Scand..

[51] McKinnon A.O., Squires E.L., Carnevale E.M., Harrison L.A., Frantz D.D., McChesney A.E., Shideler R.K. (1987). Diagnostic Ultrasonography of Uterine Pathology in the Mare. Proc. Am. Assoc. Equine Pract..

[52] LeBlanc M.M., Neuwirth L., Asbury A.C., Tran T., Mauragis D., Klapstein E. (1994). Scintigraphic Measurement of Uterine Clearance in Normal Mares and Mares with Recurrent Endometritis. Equine Vet. J..

[53] Mckinnon A.O., Squires E.L., Shideler R.K. (1988). Diagnostic Ultrasonography of the Mare’s Reproductive Tract. J. Equine Vet. Sci..

[54] Camargo C.E., Weiss R.R., Kozicki L.E., Duarte M.P., Garcia Duarte M.C., Lunelli D., Weber S., de Abreu R.A. (2013). Some Factors Affecting the Rate of Pregnancy after Embryo Transfer Derived from the Brazilian Jumper Horse Breed. J. Equine Vet. Sci..

[55] Squires E.L. (2013). Embryo Transfer Challenges and Perspectives Transferência de Embriões: Desafios e Perspectivas. Rev. Bras. Reprod. Anim..

[56] Carnevale E.M. (2008). The Mare Model for Follicular Maturation and Reproductive Aging in the Woman. Theriogenology.

[57] Uliani R.C., Ramires Neto C., Dell’Aqua J., Pessoa M.A., Camargo A.L., Alvarenga R., Alvarenga M.A. (2010). Effect of Mare Breed and Age on Embryo Transfer Efficiency. Anim. Reprod. Sci..

[58] Carney N.J., Squires E.L., Cook V.M., Seidel G.E., Jasko D.J. (1991). Comparison of Pregnancy Rates from Transfer of Fresh versus Cooled, Transported Equine Embryos. Theriogenology.

